# Le phéochromocytome surrénalien bilatéral: à propos d'un cas

**DOI:** 10.11604/pamj.2013.15.101.1931

**Published:** 2013-07-16

**Authors:** Cherif Eya, Ben Hassine Lamia, Azzabi Samira, Kaouech Zouleikha, Boukhris Imen, Kooli Chekib, Khalfallah Narjess

**Affiliations:** 1 Service de médecine interne B, Hôpital Charles Nicolle, Faculté de Médecine de Tunis, Université de Tunis El Manar, Tunisie

**Keywords:** Phéochromocytome, surrénale, scintigraphie

## Abstract

Les phéochromocytomes de localisation bilatérale sont rares. Ils s'intègrent le plus souvent dans le cadre d'une maladie familiale. Néanmoins des formes bilatérales sporadiques peuvent être observées. Ainsi, chez tout patient présentant des masses surrénaliennes bilatérales, un phéochromocytome bilatéral doit être suspecté et son caractère familial ou sporadique doit être déterminé devant le risque de récidive tumorale. A ce propos, nous rapportons l'observation d'une patiente âgée de 22 ans qui a été hospitalisée pour exploration d'une hypertension artérielle de découverte récente. L'association à des céphalées, sueurs et palpitations a orienté vers le diagnostic de phéochromocytome. Les taux urinaires des dérivés méthoxylés étaient très élevés. L’échographie et l'IRM abdominales ont noté la présence de deux masses surrénaliennes bilatérales. La scintigraphie au MIBG a montré une hyperfixation au niveau des deux surrénales, sans autres localisations. L'enquête à la recherche d'une néoplasie endocrinienne multiple ou d'une phacomatose était négative. Le traitement a consisté en une surrénalectomie bilatérale par voie cœlioscopique. L’évolution sous traitement substitutif par hydrocortisone était favorable avec une normalisation des chiffres tensionnels.

## Introduction

Le phéochromocytome est une tumeur rare développée aux dépens des cellules chromaffines, le plus souvent médullosurrénaliennes produisant un excès de catécholamines. L'atteinte surrénalienne est le plus souvent unilatérale. Mais, dans 10% des cas, une localisation bilatérale est observée [1]. Elle pose encore plusieurs problèmes relatifs à son diagnostic, aux critères de malignité et aux aspects génétiques surtout en cas d'absence d'antécédents familiaux. En effet, une mutation peut être retrouvée dans 25% des cas, même chez les patients ayant un phéochromocytome «apparemment» sporadique [2]. Les implications pronostiques de ces formes pour le patient et sa famille, dépendent des caractéristiques de chaque syndrome génétique.

## Patient et observation

**Figure 1:**
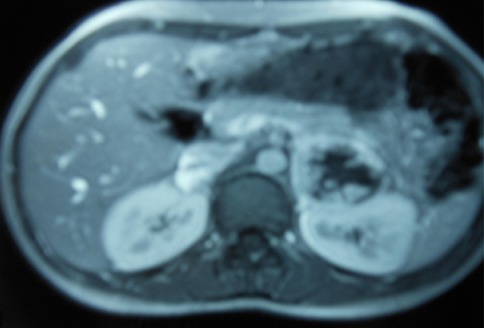
IRM abdominale: Masses tissulaires surrénaliennes bilatérales faisant 2,1cm × 1,8 cm à droite et 6,2 cm × 5 cm à gauche, hétérogène avec un rehaussement intense

**Figure 2:**
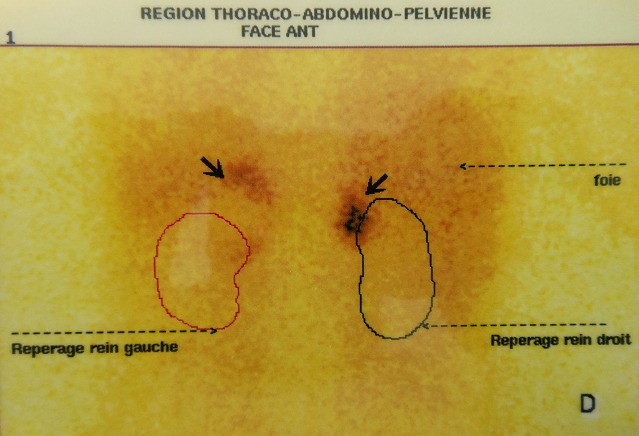
Scintigraphie à la MIBG: Présence d'une hyperfixation surrénalienne bilatérale avec absence de signe scintigraphique en faveur d'une localisation ectopique ou de métastase

Patient et observation Une patiente âgée de 22 ans, sans antécédents pathologiques notables, était hospitalisée pour exploration d'une hypertension artérielle découverte depuis 6 mois. L'examen physique était sans particularité. A la biologie, il y avait une hypokaliémie à 3,4 mmol/l et une hyperleucocytose à 12100 éléments/mm^3^. Devant la notion de troubles vasomoteurs à type de céphalée, palpitations et sueurs, un dosage urinaire des dérivés méthoxylés était pratiqué. Le diagnostic de phéochromocytome était confirmé par un taux de normétanéphrine à 135 fois la normale et un taux de 3-méthoxythyramine à 17 fois la normale. L’échographie abdominale révélait la présence de masses surrénaliennes bilatérales. L'IRM abdominale confirmait la présence de masses au niveau des deux surrénales, bien limitées, en hypersignal T2 et se rehaussant de façon intense après injection de produit de contraste. Ces masses faisaient 6,2 × 5 cm avec un compartiment nécrotique central à gauche et 2,1 × 1,8 cm à droite (Figure 1). Devant cette atteinte bilatérale, une scintigraphie à la MIBG a été pratiquée. Elle a permis de mettre en évidence des foyers d'hyperfixation au niveau des deux loges surrénaliennes (Figure 2). Par ailleurs, il n'existait pas d'autres localisations extrasurrénaliennes. Le diagnostic de phéochromocytome bilatéral était retenu. Une forme sporadique était la plus probable devant l'absence d'antécédents familiaux particuliers. L'enquête à la recherche d'une néoplasie endocrinienne multiple était négative. Une surrénalectomie bilatérale par voie cœlioscopique était pratiquée après équilibrations des chiffres tensionnels. A l'examen anatomopathologique il n'y avait pas de signes de malignité du phéochromocytome. Les suites opératoires étaient simples. L’évolution était favorable sous traitement substitutif par 30mg/j d'hydrocortisone. Avec un recul évolutif de 6 ans, aucune récidive n'a été observée.

## Discussion

Le phéochromocytome est une cause rare d'HTA (0,5 à 1%), importante à diagnostiquer du fait du risque de complications aiguës [3]. C'est une tumeur neuroendocrine qui se développe le plus souvent au niveau de la médullosurrénale. Le phéochromocytome est le plus souvent sporadique et unilatéral. Néanmoins, dans 10% des cas, il peut être bilatéral [1, 4]. Chez ces patients, une affection génétique sous jacente peut être diagnostiquée dans 80% des cas [2]. Il s'agit de néoplasie endocrinienne multiple de type 2, du syndrome de Von Hippel-Lindau ou de neurofibromatose de type 1 [5]. Un interrogatoire soigneux portant sur les antécédents familiaux et un examen physique exhaustif sont nécessaires pour reconnaître une maladie héréditaire. Mais, les antécédents familiaux peuvent être méconnus ou manquer en cas de primo-mutation [6]. Ceci justifie la réalisation systématique d'une enquête génétique dans les phéochromocytomes apparemment sporadiques, notamment en cas d’âge jeune (59% sont héréditaires) ou en présence d'atteinte multifocale (84% sont héréditaires). La découverte d'une maladie héréditaire a une grande valeur diagnostique et pronostique pour le patient et sa famille, la probabilité de récidive des phéochromocytomes familiaux étant 13 fois plus élevée que celle des phéochromocytomes sporadiques [5]. Chez notre patiente, l'interrogatoire et l'examen physique n'ont pas trouvé des signes de neurofibromatoses ou d'endocrinopathies familiales. Néanmoins, l'enquête génétique n'a pas été réalisée. En dehors de l'aspect génétique de la maladie, la découverte d'atteinte bilatérale impose la confirmation du caractère sécrétoire des deux masses surrénaliennes. En effet, l'existence d'un tableau clinico-biologique typique de phéochromocytome avec un nodule surrénalien bilatéral peut évoquer soit un phéochromocytome bilatéral soit l'association d'un phéochromocytome et d'un incidentalome d'autre nature. Dans ce cas, la scintigraphie à la MIBG a un grand intérêt pour caractériser la nature des tumeurs. Elle permet de confirmer le caractère neuroendocrine des nodules surrénaliens devant une hyperfixation bilatérale et de ce fait elle permet de proposer une sanction thérapeutique [7]. La surrénalectomie bilatérale constitue le traitement de choix. Comme chez notre patiente, un traitement substitutif est alors préconisé à vie pour éviter la survenue d'une insuffisance surrénalienne [8]. La surrénalectomie partielle préservant la corticosurrénale a été récemment proposée par certains auteurs. Elle permet d’éviter l'opothérapie et offre une meilleure qualité de vie [9].

## Conclusion

Le diagnostic de phéochromocytome bilatéral nécessite la confirmation du caractère sécrétoire des deux tumeurs surrénaliennes. La scintigraphie à la MIBG permet d'objectiver le caractère neuroendocrine des deux masses surrénaliennes et de ce fait d’éliminer un incidentalome d'autre nature. La recherche d'une affection génétique sous-jacente doit être systématique.
